# Spatial-temporal variation and risk factor analysis of hand, foot, and mouth disease in children under 5 years old in Guangxi, China

**DOI:** 10.1186/s12889-019-7619-y

**Published:** 2019-11-08

**Authors:** Huan Liu, Genxin Song, Nan He, Shiyan Zhai, Hongquan Song, Yunfeng Kong, Lizhong Liang, Xiaoxiao Liu

**Affiliations:** 1 Laboratory of Geospatial Technology for the Middle and Lower Yellow River Regions, Ministry of Education, Henan University, Kaifeng, 475004 Henan China; 2 Institute of Urban Big Data, College of Environment and Planning, Henan University, Kaifeng, 475004 Henan China; 3 The Affiliated Hospital of Guangdong Medical University, Zhanjiang, 524001 China; 4 Department of Community Health Science, Cumming School of Medicine, University of Calgary, Calgary, Canada

**Keywords:** Hand, foot, and mouth disease, GeoDetector, Socioeconomic factors, Meteorological factors

## Abstract

**Background:**

Hand, foot and mouth disease (HFMD) incidence is a critical challenge to disease control and prevention in parts of China, particularly Guangxi. However, the association between socioeconomic factors and meteorological factors on HFMD is still unclear.

**Methods:**

This study applied global and local Moran’s *I* to examine the spatial pattern of HFMD and series analysis to explore the temporal pattern. The effects of meteorological factors and socioeconomic factors on HFMD incidence in Guangxi, China were analyzed using GeoDetector Model.

**Results:**

This study collected 45,522 cases from 87 counties in Guangxi during 2015, among which 43,711 cases were children aged 0–4 years. Temporally, there were two HFMD risk peaks in 2015. One peak was in September with 7890 cases. The other appeared in May with 4687 cases of HFMD. A high-risk cluster was located in the valley areas. The tertiary industry, precipitation and second industry had more influence than other risk factors on HFMD incidence with explanatory powers of 0.24, 0.23 and 0.21, respectively. The interactive effect of any two risk factors would enhance the risk of HFMD.

**Conclusions:**

This study suggests that precipitation and tertiary industry factors might have stronger effects on the HFMD incidence in Guangxi, China, compared with other factors. High-risk of HFMD was identified in the valley areas characterized by high temperature and humidity. Local government should pay more attention and strengthen public health services level in this area.

## Background

Hand, foot, and mouth disease (HFMD) is a common epidemic disease in China. It is caused by an enterovirus and mostly occurs in children under 5 years old [1]. It is characterized by fever, oral mucosal herpes or ulcers, and skin rashes on the hands, feet, and buttocks [2]. HFMD is transmitted through respiratory droplets, saliva, and contact with infected blister fluid or feces [3]. In recent years, HFMD outbreaks have been reported frequently in Asian countries, causing widespread public health concerns [4–6].

Several studies suggested that HFMD was associated with meteorological factors such as precipitation [4, 5], humidity [7–9] and temperature [10–12], in many Asian countries, including Singapore [4], Japan [7], Vietnam [8], and China [9]. For instance, a study in Vietnam [8] found average temperature after a 5-day lag was associated with 5.6% increase in HFMD incidence. A 1-unit increase in the precipitation elevated the HFMD incidence by 0.5% on the lagged 1 and 6 days. A 1% increase in the humidity was correlated with a 1.7% increase in HFMD incidence. Studies in other cities such as Fukuoka, Japan [7], Singapore [4], and Guangzhou, China [9] also found positive relationships between average temperature and HFMD incidence. However, the association between HFMD incidence and meteorological factors, especially temperature, was regionally inconsistent [10, 11, 13]. Studies in Tokyo [13], Beijing [11], and Shandong [10] found a non-linear association between average temperature and HFMD incidence. One study in Shandong, China, found a decreasing trend between temperature and HFMD incidence when the average temperature was above 21 °C [10]. Conversely, the study in Beijing observed an increasing trend in the HFMD incidence along with rising temperature, having the largest association observed at 25.0–27.5 °C [11].

The HFMD incidence is affected not only by meteorological factors, but also by socioeconomic conditions [14, 15]. Previous study found that high population density of children under 9 years old and tertiary industry were the primary risk factors in HFMD incidence [15]. A study in Beijing, China also found that urban areas with higher population density and stronger population mobility suffered a higher HFMD incidence than rural areas [16]. A Vietnam study suggested that children with better socioeconomic conditions (living in permanent houses and having access to safe water) had significantly lower rates of contracting HFMD, compared to those without such conditions [14].

Located in the south of China, Guangxi had experienced serious HFMD epidemics with an average annual HFMD incidence of 361.13/100,000 in recent year (2008–2015) [17, 18]. Guangxi ranked first among all provinces in China in 2015 in terms of HFMD mortality and second in terms of HFMD incidence [17]. Previous studies on HFMD in Guangxi have mainly focused on spatiotemporal variations [19], epidemiological characteristics [11, 20] of HFMD cases and the association between meteorological factors and HFMD incidences. To the best of our knowledge, there is limited evidence on the association between socioeconomic factor and HFMD in Guangxi. Understanding the association between socioeconomic factors, meteorological factors, and their joint effect on HFMD is very important for developing effective interventions on HFMD. In this study, we aim to identify the areas with higher HFMD risk using spatiotemporal analysis. Then we examine the association between socioeconomic, meteorological factors and their join effect on HFMD in Guangxi, China.

## Methods

### Study area

**Fig. 1:**
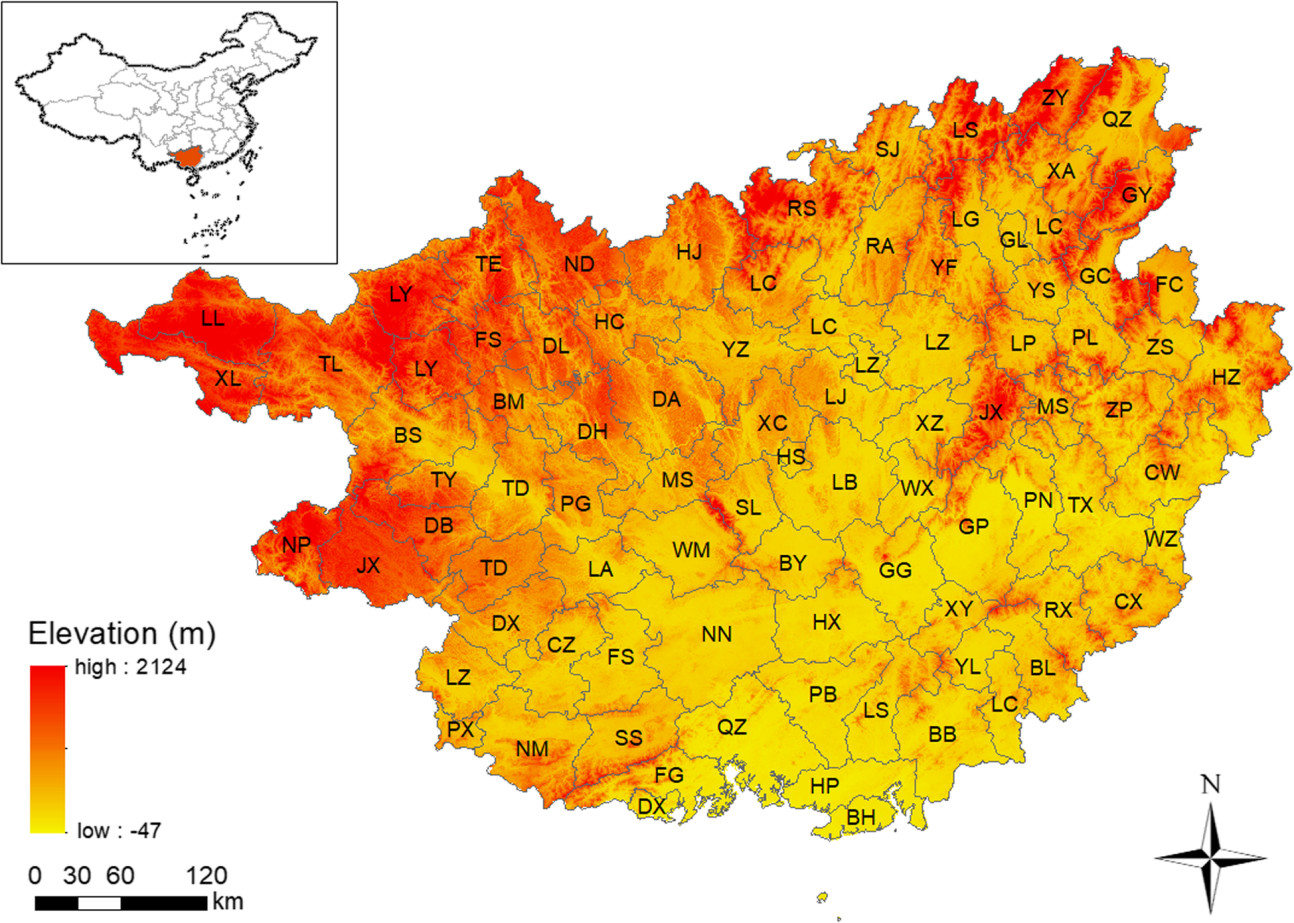
Geographical location of study area, Guangxi Zhuang Autonomous Region, China. (The author drew this map by ArcGIS 10.3 software). Note: Refer to Additional file 1: Table S1 for the full name of the county

Located between latitude 20°54′-26°24′ north and longitude 104°26′-112°04′ east, Guangxi Zhuang Autonomous Region is a coastal province in the southern China adjacent to the Beibu Gulf (Fig. 1). Covering 23.67 km^2^, Guangxi has a typical subtropical monsoon climate with warm climate and abundant rain. It is dominated by mountains, with a landform inclined from high in the northwest to a low basin in the southeast. It comprises 111 counties belonging to 14 regions (municipal districts). In this study, the research area was divided into 87 areas by merging municipal districts. Guangxi has a population of approximately 52 million in 2015, among which about 3.93 million were children under 5 years of age.

### Data sources

We obtained the HFMD-related inpatient medical records from January 1, 2015 to December 31, 2015 in Guangxi collected by the Guangdong Medical University. These records provided information on the disease classification, admission data, length of stay, age, residential location, surgical conditions and diagnostic code. Based on the International Classification of Diseases (ICD-10) codes, we identified HFMD cases as those with Enteroviral vesicular stomatitis with exanthem (B08.4) [21]. According to the clinical diagnosis, the HFMD cases were diagnosed as either ordinary cases or severe cases. The former had the symptoms of fever symptoms by a vesicular rash [22], while the latter had the symptoms of encephalitis, aseptic meningitis, or polio-like paralysis [23]. This study was reviewed and approved by the Ethics Committee of Henan Medical School.

We aggregated the HFMD-related inpatient records for each day at the county level based on patient’s residential location. Nanning and Beihai were not included in this study due to the lack of HFMD data in children’s hospital. Considering that the vast majority of HFMD admission cases were children, this study only included patients under 5 years old which accounted for 96% of the total number of cases. The HFMD incidence in each county was calculated as the ratio of HFMD cases to the background population of children under 5 years old.

Meteorological data were obtained from the National Meteorological Information Center [24], including daily temperature, daily average relative humidity, and daily average precipitation. Topographic elevation data were acquired from the Geospatial Data Cloud [25].

Socioeconomic data were collected from the Guangxi Statistical Yearbook [26] and the Sixth National Census [27], including land area, permanent population, gross domestic products (GDP), proportion of primary industry, proportion of secondary industry, and proportion of tertiary industry in 87 counties in Guangxi. The primary industry includes agriculture, forestry, animal husbandry, and fisheries. The secondary industry is mainly manufacturing. The tertiary industry refers to circulation department and service department. The population of children under 5 years old in 2010 was included in this analysis because of the availability of the National Census in 2010 instead of 2015.

### Risk factors

In this study, gross population density and population density of children under 5 years old were adopted as risk factors. Population density was calculated by dividing the population of the county by the area of the county. Through collinearity diagnosis (Additional file 2: Table S2 and Additional file 3: Table S3), daily precipitation, daily average relative humidity, and daily average temperature were selected as the meteorological conditions [19]. GDP and industrial structure were adopted as the socioeconomic conditions [20]. Topographic elevation was also listed as a risk factor given the preliminary descriptive analysis showing that HFMD incidence was higher in the basin than in other areas. In total we adopted 10 risk factors of HFMD incidence including daily precipitation, daily average relative humidity, daily average temperature, elevation, gross population density, population density of children under 5 years old, GDP, proportion of primary industry, proportion of secondary industry, and proportion of tertiary industry.

### Spatial autocorrelation analysis

We applied spatial autocorrelation methods, including global Moran’s *I* and local Moran’s *I* statistics to describe and plot spatial clusters and regions of HFMD distribution in ArcGIS 10.3 software (https://www.esri.com) [1, 19]. The Moran’s *I* statistic is calculated by Eq. (1)

1 $$ I=\frac{N}{S_O}\;{\sum}_i\;{\sum}_j{w}_{ij}\;\frac{\left({x}_i-u\right)\;\left({x}_j-u\right)}{\sum_i{\left({x}_i-u\right)}^2} $$

where *N* is number of areas; *x*_*i*_ and *x*_*j*_ are observations for areas *i* and *j* with mean *u*; *w*_*ij*_ is the element in the spatial weight matrix corresponding to the observation pair *i, j*; *S*_*o*_ is the sum of *w*_*ij*_. The values of Moran’s *I* index range from − 1 to + 1. A Moran’s *I* index close to − 1 indicates clustering, whereas a Moran’s *I* index close to 1 indicates dispersion. In general, Moran’s *I* values can be converted to *Z*-score, where the *Z*-score > 1.96 or < 1.96 indicate spatial autocorrelation significant at the 95% confidence level. Moran’s *I* > 0, *Z* > 1.96 and *p* < 0.05, indicating local spatial aggregation in the distribution of HFMD.

Unlike global Moran’s *I* with an assumption of spatial stationarity, the Local Moran’s *I* enables the assessment of significant local spatial clustering around an individual location. Local Moran’s *I* calculates Local Moran’s *I* index for each county which indicating the extent of significant difference between the county of interest and its neighbors within a predefined neighboring context. In this study, we applied Inverse Distance to define the neighboring context. We assigned a weight of 1 to those neighbors located inside the neighboring context and a weight of 0 to those outside the neighboring context.

### GeoDetector model

GeoDetector is a spatial variance analysis based on statistics, which quantitatively expresses the spatial stratified heterogeneity of the research object by analyzing the differences and similarities of intra-layer and inter-layer variance (http://www.geodetector.org/) [15, 28]. By dividing the study area into subregions, the model compares the sum of the variances of subregions and the variance of the region as a whole. If the former is smaller than the letter, the model assumes there is a spatial differentiation [29–31]. If the spatial distribution of the two variables tends to be consistent, there is a statistical correlation between them. The GeoDetector requires the independent variable to be categorical, so the independent variable needs to be discretized. The explanatory powers and the explanatory powers of interactive calculated by GeoDetector model were used to assess the effectiveness of different discretization [32]. The higher the explanatory powers and the explanatory powers of interactive, the better the discretization method. The natural breakpoint method minimizes the data difference in the same category and maximizes the difference between categories. In this study, we adopted eight layers with the highest explanatory powers by using the natural break breaking method.

#### Factor detector

The explanatory power for the incidence rate of HFMD is calculated by using variance and total variance of each spatial partition [33] to identify the main factors affecting the HFMD. The explanatory power is measured by *q*, and the formula for calculating *q* is shown in Eq. (2) [34].

2 $$ {\displaystyle \begin{array}{c}q=1-\frac{\sum \limits_{h=1}^L{N}_h{\sigma^2}_h}{N\;{\sigma}^2}=1-\frac{SSW}{SST}\\ {} SSW=\sum \limits_{h=1}^L{N}_h{\sigma_h}^2, SST=N{\sigma}^2\end{array}} $$

Where *h* is the number of layers of the dependent variable *Y* or the independent variable *X*; in this paper, the number of layers is 8. *N* and *N*_*h*_ are the number of units in layer *h* and in the entire study area, respectively. *σ*^*2*^ denotes the variance of *Y*, and *σ*_*h*_^2^ is the variance of the *Y* in stratum *h*. *SSW* and *SST* denote the within sum of squares and total sum of squares, respectively. The value of *q*∈[0,1]. The larger the value of *q*, the greater the influence of this factor on the incidence of HFMD.

#### Risk detector

It is used to calculate the average incidence rate in different areas. Greater average incidence means greater danger to the health of people with the area. The *t* statistics were used to determine whether there is a significant difference in the mean of incidence between two subregions.

#### Interaction detector

By identifying the interactions between different risk factors *X*_*1*_ and *X*_*2*_, interaction detector shows whether the interaction will increase or decrease the explanatory power of the dependent variable *Y*. The two layers *X*_*1*_ and *X*_*2*_ are overlaid and their attributes were combined as a new attribute *X*_*3*_. By comparing the value of q of *X*_*1*_*, X*_*2*_ and *X*_*3*_, we are able to determine the influence of the interaction [35]. The interaction relationships are cataloged as follows:

- Enhance: if q(X_1_∩X_2_) > q(X_1_) or q(X_2_)
- Enhance, bivariate: if q(X_1_∩X_2_) > q(X_1_) and q(X_2_)
- Enhance, nonlinear: if q(X_1_∩X_2_) > q(X_1_) + q(X_2_)
- Weaken: if q(X_1_∩X_2_) < q(X_1_) + q(X_2_)
- Weaken, univariate: if q(X_1_∩X_2_) < q(X_1_) or q(X_2_)
- Weaken, nonlinear: if q(X_1_∩X_2_) < q(X_1_) and q(X_2_)
- Independent: if q(X_1_∩X_2_) = q(X_1_) + q(X_2_)

## Results

### Spatial and temporal variations of HFMD

**Fig. 2:**
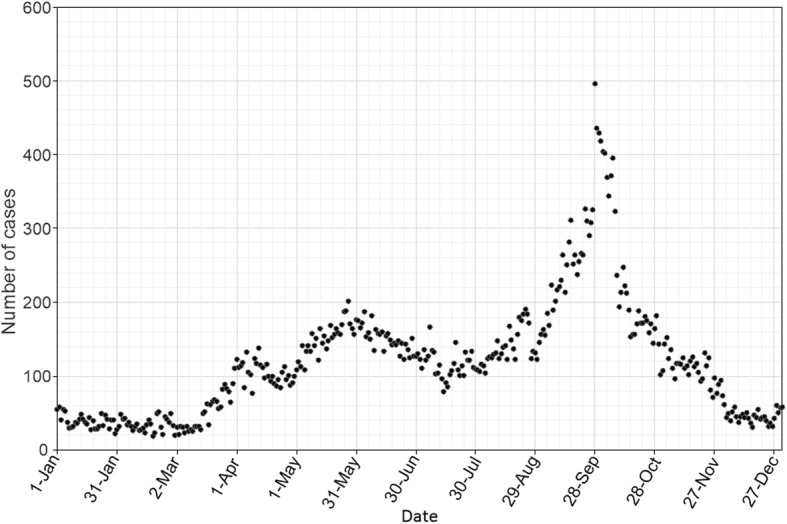
Daily cases of HFMD in Guangxi, China in 2015. Note: Not including Nanning and Beihai

We identified 45,522 cases in 2015, among which 43,711 cases were children under 5 years old accounting for 96% of the total incidence. The incidence rate of children under 5 years old was 1440.99/100,000, compared to the incidence rate of 95.88/100,000 among the general population. As shown in Fig. 2, the HFMD cases had substantial seasonal differences. One peak was in May with 4687 cases of HFMD. The second peak was in September with a maximum of 7890 cases. The lowest number of cases was observed in winter.

**Fig. 3:**
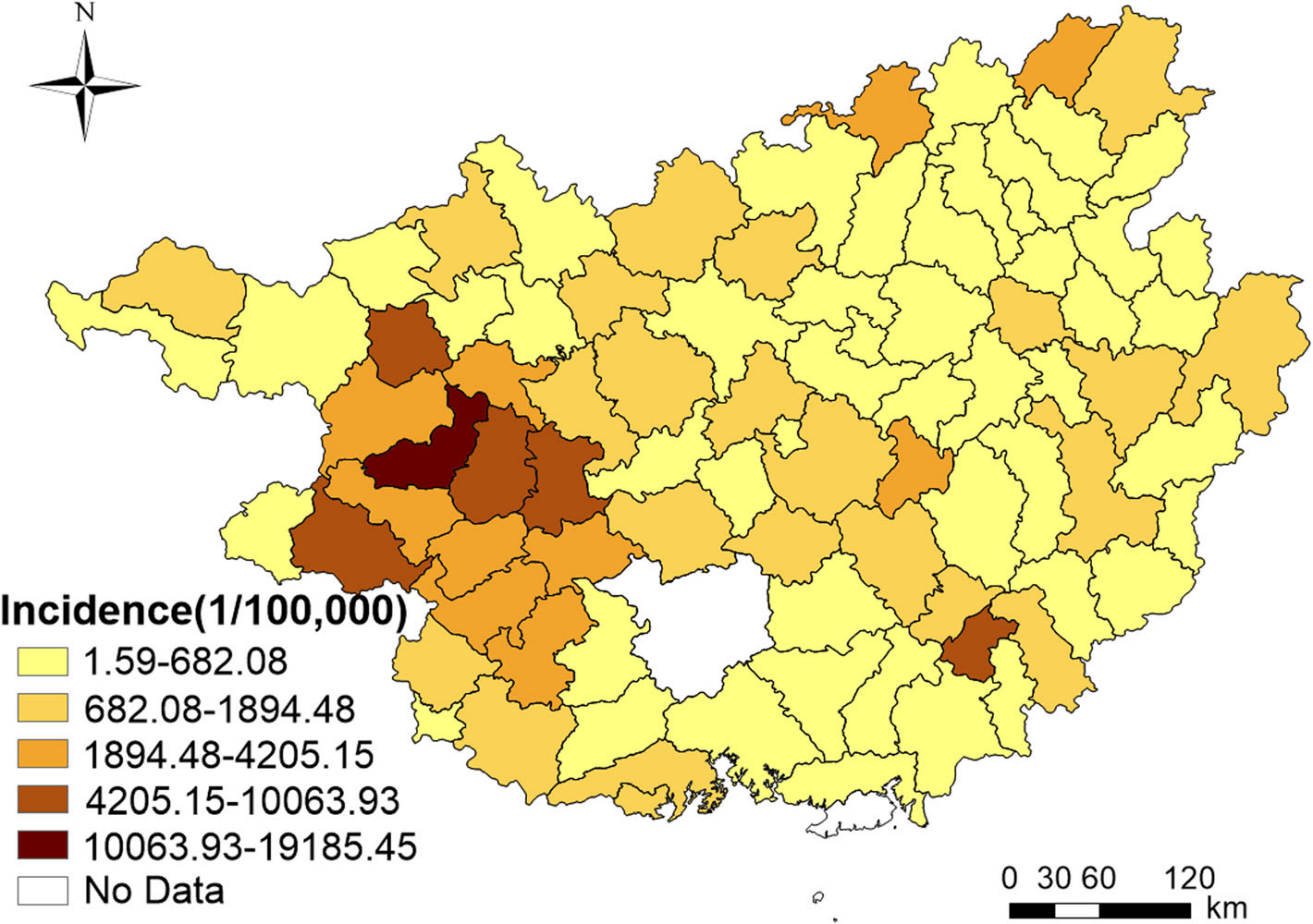
HFMD among children aged 0–5 years in 87 counties of Guangxi in 2015. (The author drew this map by ArcGIS 10.3 software)

**Fig. 4:**
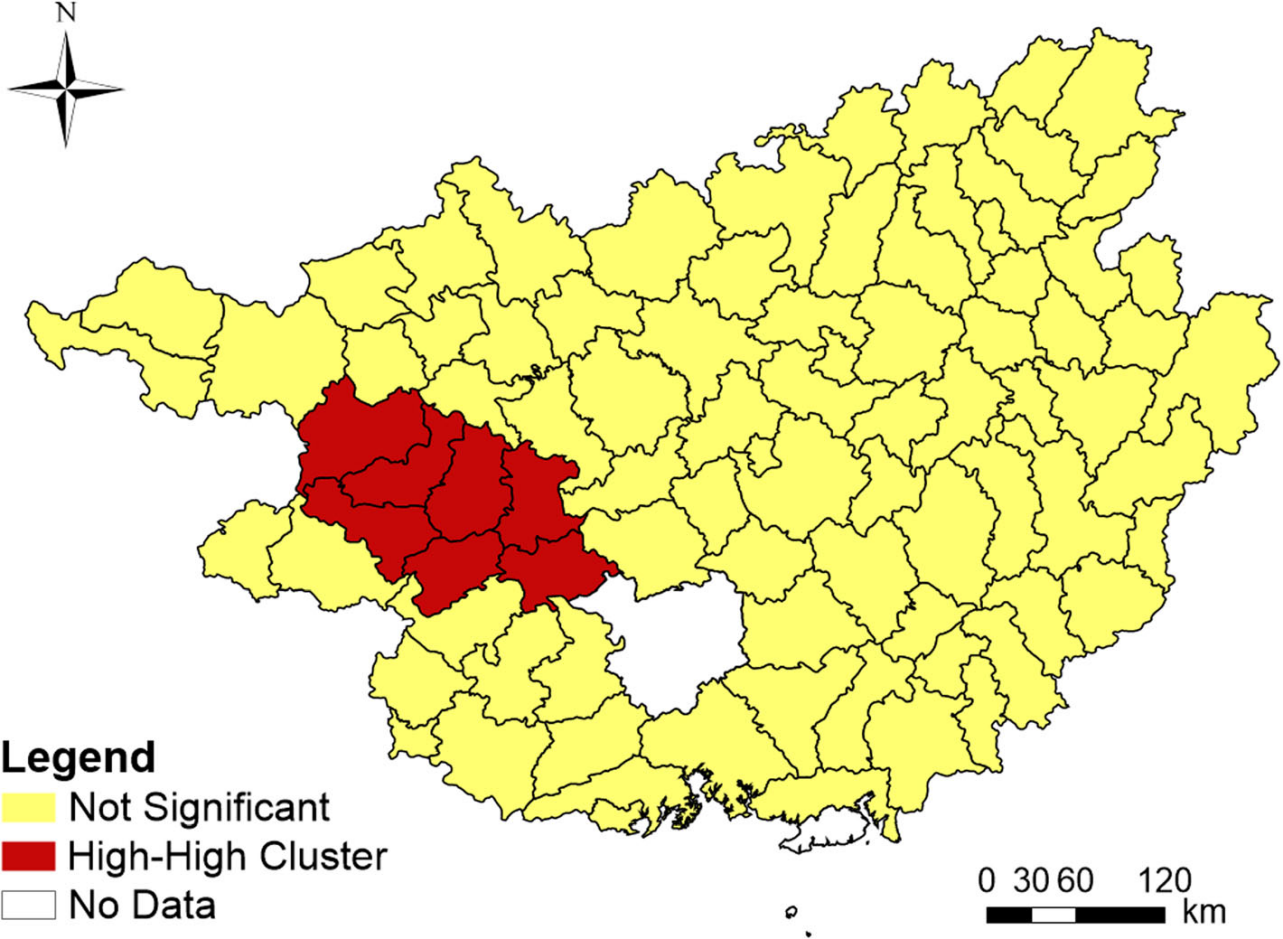
Local Moran’s I of the incidence of HFMD in Guangxi. (The author drew this map by ArcGIS 10.3 software)

Figure 3 presented an obvious spatial variation in the incidence rates of HFMD among children under 5 years old in 87 counties of Guangxi in 2015. The highest HFMD incidence rate was observed in Tianyang county (19,185.45/100,000) and the lowest in Rong county (1.59/100,000). The global Moran’s *I* index of the incidence rate of HFMD was 0.314 and the corresponding Z value was 4.636. As the Z value was significantly higher than the critical Z value of 1.96 at the significance level of *p* = 0.05, it indicated that the incidence of HFMD had a statistically significant spatial autocorrelation [19, 36, 37]. Local Moran’s *I* identified 7 counties clustered in the west of Guangxi (Tianyang, Tiandong, Baise, Debao, Tian’an, Pingguo and Longan), having statistically higher HFMD incidence than the surrounding counties (Fig. 4).

### Descriptive analysis

A simple statistical description of risk factors is given in Table 1. With an average GDP of 1528 × 10^7^ CNY ranging from 196 × 10^7^ CNY to 16,886 × 10^7^ CNY, there was a great variance in the distribution of wealth. The distribution of population density also differed greatly for the gross population density ranging from 41.68 person/km^2^ to 1611.64 person/km^2^ and for the population density of children under 5 years old ranging from 2.06 person/km^2^ to 79.28 person/km^2^. The population was mainly distributed in economically developed coastal cities such as Guilin, Yulin, etc. The average proportion of primary industry, secondary industry, and tertiary industry were 23.5, 40.98 and 35.52%, respectively, indicating the difference in the industrial structure in Guangxi. Pingxiang had the highest proportion of tertiary industry (62.39%) among the counties, while Yongfu had the least (17.37%).

**Table 1:** Statistical description of incidence and potential risk factors of HFMD

Variables	Mean	Min	Max	25%	50%	75%
Incidence (1/100,000)	1440.99	1.59	19,185.45	146.39	556.08	1492.35
PRE (mm)	53.31	37.45	78.23	48.22	53.64	56.90
RHU (%)	80.48	76.26	83.94	79.06	80.83	81.57
TEM (°C)	21.10	17.74	23.36	19.89	21.33	22.53
POP (person/km^2^)	219.82	41.68	1611.64	115.85	162.59	244.42
POP_4 (person/km^2^)	16.73	2.06	79.28	8.21	12.01	20.09
GDP (10^7^CNY)	1528.09	196.29	16,886.57	559.04	1081.77	1901.63
Primary_indu (%)	23.50	0.88	42.18	18.07	23.59	29.01
Secondary_indu (%)	40.98	17.16	65.84	33.46	40.92	49.34
Tertiary_indu (%)	35.52	17.37	62.39	28.51	35.93	40.76

Not including Nanning and Beihai

*PRE* Precipitation, *RHU* Relative humidity, *TEM* Temperature, *POP* Gross population density, *POP_4* Population density of children under 5 years old, *GDP* Gross domestic product, *Primary_indu* Proportion of primary industry, *Secondary_indu* proportion of secondary industry, *Tertiary_indu* Proportion of tertiary industry

**Fig. 5:**
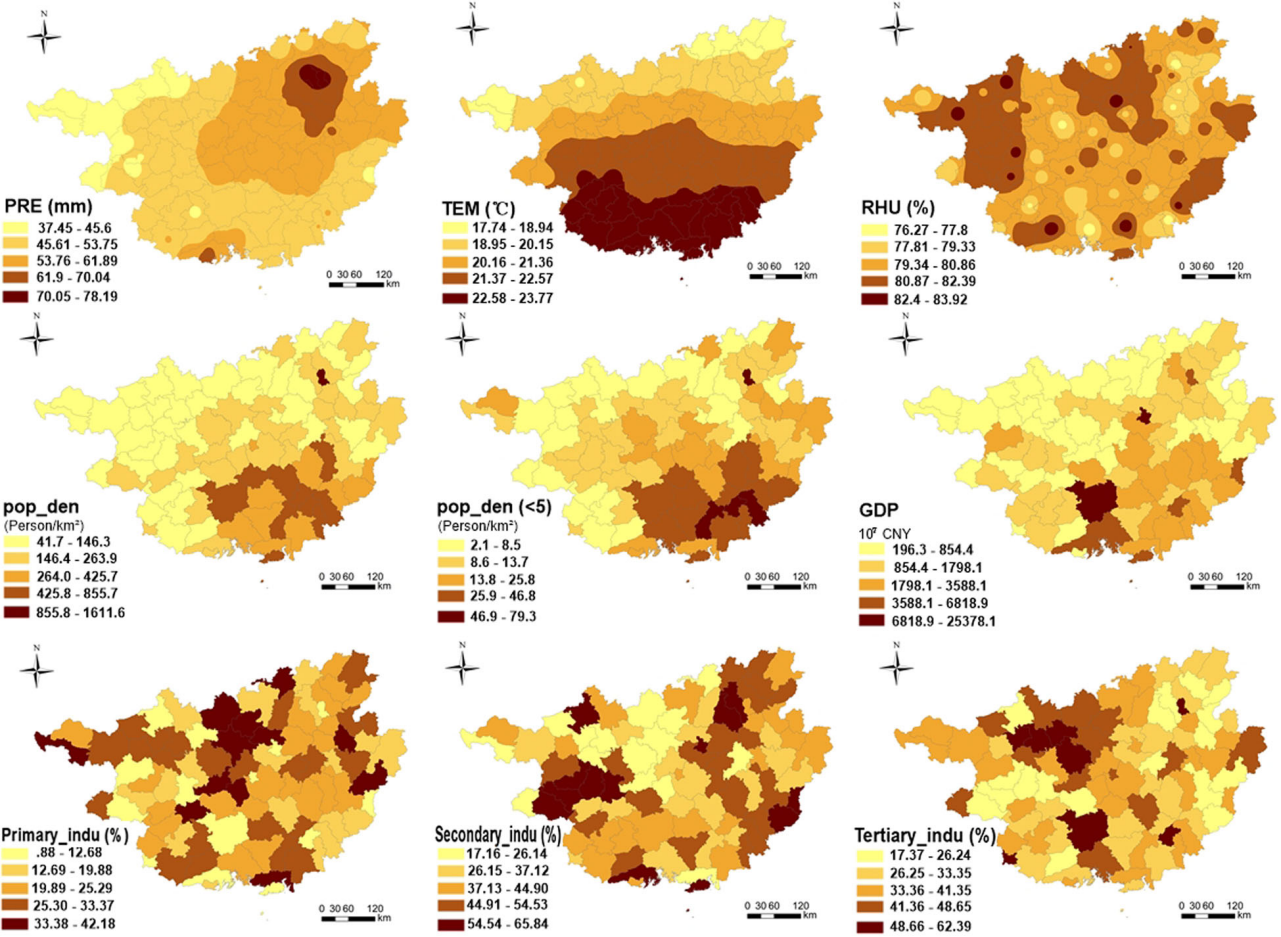
Geographical variations of the risk factors for HFMD in Guangxi. (The author drew this map by ArcGIS 10.3 software)

There were also differences in meteorological factors between counties (Fig. 5). Guangxi had abundant precipitation especially in the northeast areas such as Guilin and Lingui, where the daily precipitation could exceed 53 mm. Under the influence of latitude, the temperature gradually increased from north to south. Affected by the meteorological factors and topography, the relative humidity had little difference and generally remains at a relatively high level with the lowest relative humidity of 76%.

### Factor detector analysis

As shown in Table 2, the determinant power of the proportion of the tertiary industry was obviously associated with transmission of HFMD (*q* = 0.24), indicating that the tertiary industry mainly explains the spatial heterogeneity of the incidence of HFMD. Precipitation, secondary industry, gross population density, temperature and GDP were also associated with the spread of HFMD, having explanatory powers *q* of 0.23, 0.21, 0.12, 0.09 and 0.08 respectively.

**Table 2:** Explanatory power of each impact factor on the incidence of HFMD

	Tertiary_indu	PRE	Secondary_indu	POP	TEM	GDP	Primary_indu	POP_4	RHU	GEO
q statistic	0.24	0.23	0.21	0.12	0.09	0.08	0.05	0.05	0.04	0.02
*p* value	0.00	0.00	0.00	0.00	0.00	0.00	0.00	0.00	0.00	0.00

*GEO* Elevation, *Primary_indu* Proportion of primary industry, *Secondary_indu* Proportion of secondary industry, *Tertiary_indu* Proportion of tertiary industry

### Risk detector analysis

**Fig. 6:**
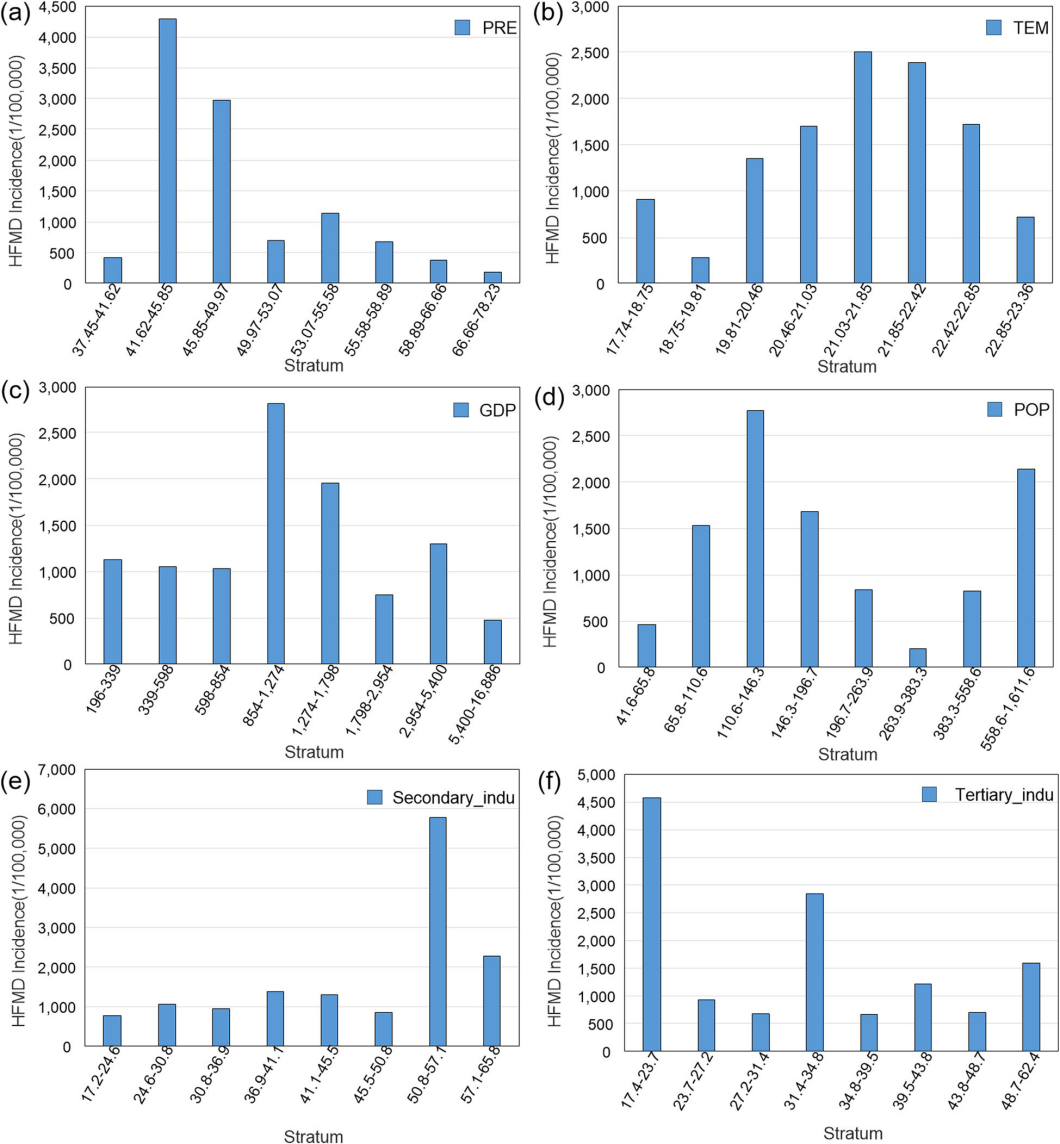
Average incidence of HFMD according the different risk factors stratums. **a** Precipitation; **b** Temperature; **c** GDP; **d** Gross population density; **e** Proportion of secondary industry; **f** Proportion of tertiary industry

Figure 6 showed the effect of different risk factors on the incidence rate of HFMD. We found that when the daily average precipitation exceeded 45.9 mm, the incidence of HFMD decreased. There was an inverted V-shape association between temperature and HFMD. When temperature was 21 °C, the HFMD reached a peak. Risk detector revealed an inverted U-shape association between GDP and HFMD. The incidence of HFMD increased along with GDP, reached a peak when the GDP was at 1274 × 10^7^ CNY and then decreased afterwards. Similar pattern was observed for the association between the population density of children under 5 years old and HFMD. With regards to the association between industry structure and HFMD, the incidence of HFMD was the highest when the proportion of secondary industry was between 50 and 57% and when the proportion of tertiary industry was around 20, respectively.

### Interaction detector analysis

**Fig. 7:**
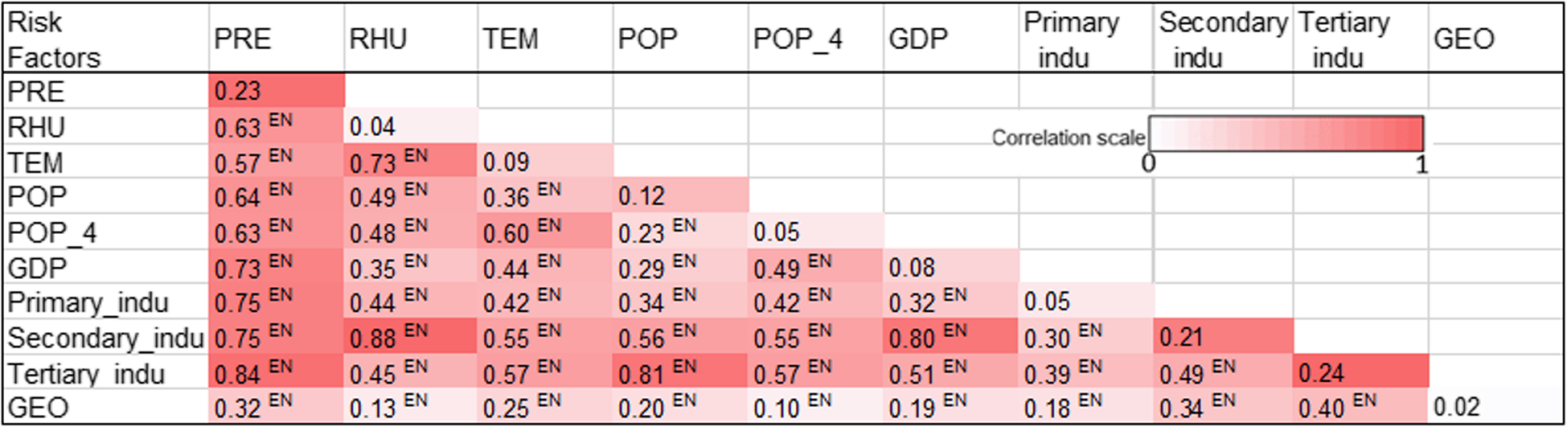
The value of q for interactions between pairs of factors on the incidence of HFMD. Note: EN: Enhance (nonlinear)

The study found that the interaction of any two risk factors has greater explanatory power than any single risk factor, especially for those interactive effect among socioeconomic factors and metrological factors. Compared with their individual impact, they all presented the effect of “nonlinear enhance”. As shown in Fig. 7, the q statistics of tertiary industry was 0.24, which was increased to 0.81 after accounting for the interactive effect of precipitation on the HFMD incidence. As 0.81 is significantly higher than the sum of 0.24 (q statistics of tertiary industry) and 0.23 (q statistics of precipitation), the result suggested that tertiary industry and precipitation has a significantly enhanced nonlinear interactive associations on the incidence rate of HFMD. The explanatory power of tertiary industry increased to 0.57 after considering the interactive effect of temperature on HFMD incidence.

Similar as the explanatory power of tertiary industry, after accounting for the effects of precipitation (*q* = 0.23) and temperature (*q* = 0.09) on the incidence of HFMD, the explanatory power of secondary industry increased from 0.21 to 0.75 and 0.55 respectively, and of gross population density increased from 0.12 to 0.64 and 0.36 respectively. The interaction of these risk factors could effectively explain the spatial heterogeneity of the HFMD, and the selected risk factors had a tendency of strengthen interaction.

## Discussion

To the best of our knowledge, there is limited evidence on the associations between socioeconomic factors and the incidence of HFMD in Guangxi China. In this study, we explored the spatial and temporal pattern of HFMD incidence and further investigated the effects of socioeconomic factors and its interactive effects with metrological factors on the HFMD incidence. We have three important findings. First, there was a difference in the timing of peak for the outbreak of HFMD incidence among different areas. Second, we identified counties having significant high-risk of HFMD incidence located primarily in the valley region over the study area, where both economic development level and population density were at a lower level. Third, we identified the statistically significant effects of tertiary industry, secondary industry and gross population density on the HFMD incidence. Most importantly, the explanatory power of each socioeconomic factors was greatly increased after accounting the interactive effects of meteorological factors.

Our finding on the temporal pattern of HFMD incidence was aligned with previous findings in studies in Guangdong [38], Jiangsu [39], Sichuan [40], Shandong [41], Guangxi [42, 43] China. However, some other studies reported a different timing of HFMD outbreak. We found that the highest peak was in September and the second-highest peak was in May in Guangxi, while the peak of HFMD in Beijing was observed in June [16] and the HFMD peak in Shandong appeared in July [10]. A potential reasoning was that the differences in temperature, precipitation and humidity among different regions resulted in the difference in the seasonal variation of HFMD. In addition, we found that HFMD mainly occured in children under 5 years old, which was consistent with other research in China [10].

In the spatial dimension, we found that significant high-risk cluster of HFMD incidence was mostly located in valley area, where both economic development level (less than 50th percentile, GDP < 1081.77 × 10^7^ CNY) and population density (less than 40th percentile, the under-5 population density < 10.68 person/km^2^) were at a lower level. Previous studies indicated that high HFMD incidence were mainly concentrated in the urban areas [15, 16, 44] or their surrounding counties [45] due to the large numbers of people being conductive to the spread of HFMD. However, in this study, high-risk cluster didn’t appear in large cities with high GDP and high population density, but in valley areas underdeveloped economic and low population density. One possible explanation was that people living in this valley area have low economic income, fewer opportunities to obtain an education and lack awareness of disease prevention. In addition, these counties have imperfect medical infrastructure, low level of health services, and lack of appropriate medical diagnosis and disease treatment [45]. From the perspective of meteorological conditions, these high-risk HFMD areas were located in the middle of the Youjiang River valley, which is famous for being a natural big greenhouse [46]. With an average annual temperature of 22 °C and annual precipitation of 1100 mm, the warm weather and high precipitation could facilitate the survival and transmission of the HFMD enteroviruses [47, 48]. Most importantly, as shown in the interactive analysis, the interactive effects of socioeconomic and meteorological factors on the incidence of HFMD were greatly enhanced when considering the factors simultaneously.

With regards to the socioeconomic conditions, we identified the effects of tertiary industry and secondary industry on the incidence of HFMD. Several studies have reported that socioeconomic plays an important role in HFMD, such as Henan [45], Sichuan [44] and Beijing-Tianjin-Hebei [15, 16, 49]. In this study, the tertiary industry was crucial risk factor on HFMD which was consistent with previous studies [15, 50]. Tertiary industry was characterized by floating population, which might increase contacts among population and accelerate the spread of the virus [15]. The association between secondary industry and the incidence of HFMD was supported by previous studies showing that the secondary industry had an important contribution to the risk of HFMD incidence [51, 52]. Increasing industrial activity may exacerbate air pollution. It is believed that living in an environment with air pollution can cause serious damage to the respiratory system [51]. The more serious the respiratory damage is, the more susceptible the HFMD is infected among vulnerable population, especially in children due to their immature immune system that are more sensitive to air pollution [52].

The nonlinear association between temperature and HFMD observed in this study was consistent with previous studies [10, 11, 53]. We found that the temperature was around 21 °C, HFMD incidence peaked (Fig. 6), which was similar as the study in Shandong [10]. Xu proposed that a temperature range of 25.0–27.5 °C was expected to generate the highest relative risk for HFMD in Beijing [11]. It may be caused by the warm environment which is good for outdoor activities and the reproduction and spread of the virus [44]. However, in extremely hot environment, children may tend to stay in an air-conditioned environment, which would reduce the risk of HFMD [20, 54].

We found that when the daily precipitation exceeded 45.9 mm, the incidence of HFMD decreased. The result was similar to the findings from previous studies [4, 20]. Wang found that extreme precipitation (precipitation > 14.85 mm) was association with a reduced HFMD [20]. Warm and humid environment was suitable for the breeding and prevalence of the virus, but heavy downpour might damage the environment in which the virus survived [20]. And heavy downpour reduced the contact between children, resulting in a decreased incidence of HFMD.

Our research had some strengths. First, this research filled the gap in the knowledge on the associations between socioeconomic factors and HFMD incidence in Guangxi China, and the associations may be greatly enhanced by accounting for the interactive effects of meteorological factors. Second, we identified clusters of HFMD incidence in less developed areas, which was different from other studies reporting high HFMD incidence in urban areas. At the same time, our research had some limitations. First, the time span was only 1 year, which might not adequately represent the real pattern of HFMD onset. Secondly, we ran the spatial analysis using districts and counties as the basic analysis unit, which was not able to capture more detailed local information. Third, the outpatient visits were not considered in this study due to the lack of data availability. However, the estimated HFMD prevalence using inpatient admission records is the best estimate possible representing the patients with severe HFMD under 5 years old for the whole Guangxi, China. Finally, the problem of how to select the appropriate classification algorithm and divide the data into several layers remains to be studies. Further studies should be conducted to examine the effect of individual behavioral and air quality on the incidence of HFMD.

## Conclusions

This study found that high-risk area of HFMD was located in valley areas in Guangxi, also known to have relatively depressed socioeconomic conditions and low population density. Local authority should pay more attention to valley areas with low economic and population density, and allocate more medical resources in this area.

## Supplementary information

**Additional file 1: Table S1.** Abbreviations of counties’ name. In order to clearly recognize the location of each county in Fig. 1, we made this comparison table of abbreviations of counties’ name.

**Additional file 2: Table S2.** Collinearity Diagnostics. According to the results of Collinearity diagnostics, the maximum condition index is 512.831, which is greater than 50. And the variance proportions of temperature, the highest temperature and the lowest temperature are 99, 60 and 62%. There is multicollinearity between these variables.

**Additional file 3: Table S3.** Collinearity statistics. A multicollinearity evaluation of these meteorological factors (including daily average temperature, daily average relative humidity, hot, cold and extreme humid) shows that the VIF are more than 10 units, indicating that the degree of confounding effects of the climate factors is not acceptable. So, we did not analyze the effect of extreme situation on the HFMD.

## Data Availability

The meteorological data used in the study were available from National Meteorological Information Center (http://data.cma.cn/). The socio-economic data mainly came from Guangxi Statistical Yearbook (http://www.gxtj.gov.cn/). The Sixth National Census came from CNKI (http://data.cnki.net/). The datasets of HFMD generated during and analysed during the current study are not publically available due to confidentiality requirements but are available from the corresponding author on reasonable request.

## Abbreviations

CNY: Chinese Yuan
EN: Enhance (nonlinear)
GDP: Gross domestic product
GEO: Elevation
HFMD: Hand, foot and mouth disease
ICD-10: International Classification of Diseases
POP: Gross population density
POP_4: Population density of children under 5 years old
PRE: Precipitation
Primary_indu: Proportion of primary industry
RHU: Relative humidity
Secondary_indu: Proportion of secondary industry
TEM: Temperature
Tertiary_indu: Proportion of tertiary industry
